# Capacitive and Sensing Responses of Biomass Derived Silver Decorated Graphene

**DOI:** 10.1038/s41598-019-56178-4

**Published:** 2019-12-23

**Authors:** Rabina Bhujel, Sadhna Rai, Khanindram Baruah, Utpal Deka, Joydeep Biswas, Bibhu P. Swain

**Affiliations:** 1 Centre for Materials Science and Nanotechnology, Sikkim Manipal Institute of Technology, Sikkim Manipal University, Majhitar, Rangpo-737136 East Sikkim India; 2 Department of Chemistry, School of Physical Sciences, Sikkim University, Tadong, Gangtok, Sikkim 737102 India; 3 Department of Physics, Sikkim Manipal Institute of Technology, Sikkim Manipal University, Majhitar, Rangpo-737136 East Sikkim India; 4 Department of Chemistry, Sikkim Manipal Institute of Technology, Sikkim Manipal University, Majhitar, Rangpo-737136 East Sikkim India; 5 Department of Physics, National Institute of Technology, Langol, Manipur India

**Keywords:** Materials science, Nanoscience and technology

## Abstract

A new, easy and green method is utilized for producing silver decorated graphene for its application in sensors and supercapacitors. The biomass-derived silver decorated graphene (AgGr) samples are prepared using an APCVD reactor with varying the process temperature from 600 to 800 °C. The as-synthesized AgGr samples were then characterized by AFM, SEM, Raman spectroscopy, FTIR spectroscopy, XRD, cyclic voltammetry and impedance spectroscopy. The interlayer spacing and I_D_/I_G_ ratio of the AgGr samples varied from 3.6 to 3.7 Å and 0.87 to 1.52, respectively, as the process temperature was raised from 600 to 800 °C. The SEM image shows the distribution of the flower-like structure of Ag flakes in the graphene sheet for the AgGr-800 sample. Also, the greater number of active sites on the surface of AgGr-800 and the presence of a higher number of defects makes it least useful for p-nitrophenol sensing due to the excess opening of the CV curve but has a maximum capacitance of 93.5 Fg^−1^ in 1 M H_2_SO_4_. AgGr-600 showed extremely good sensing of p-nitrophenol than the other AgGr samples. Therefore this novel technique can be utilized for the large scale manufacture of various metal decorated graphene samples for their application in different fields.

## Introduction

Graphene is a transparent sheet-like structure with the two-dimensional network of sp^2^ hybridized carbon arranged in a honeycomb structure. The excellent properties of graphene make it extremely important for its application in various fields like supercapacitors^1^, sensors^2^, solar cells^3^, various electronic and photonic devices^4,5^, etc. Graphene sheets are already proven to have very good sensing properties for various toxic and harmful chemicals due to their porous nature with good adsorption properties. Moreover, the high electrical conductivity of this material favors very good supercapacitive behavior in different electrolytes. Therefore recently various techniques are employed by the researchers across the world in order to obtain better results by incorporating some new features to this graphene sheet. One of the common methods is the incorporation of metal nanoparticles in the graphene sheet. Metal nanoparticles have drawn huge interest from the researchers due to their potential applications in various active fields. Among the different metal nanoparticles, silver nanoparticles (AgNPs) are one of the most commonly used metal nanoparticles due to their superior electrical, electrochemical and sensing properties than the other metal nanoparticles^6^. The decoration of such metal nanoparticles on the graphene sheet can enhance the properties of the graphene sheet to many fold times with their applications in various fields^7–9^. Silver decorated graphene is one of the most widely used materials in different fields like sensing, energy-storage, medicines and also in industries due to their tremendous chemical and physical properties. The extremely good electrical and electrochemical properties allow their extensive applications for supercapacitors as well as for the sensing of various toxic chemicals. The fabrication of chemical sensors has recently become an important area of research due to the increasing environmental pollution, which is the main cause of the spreading of many life-threatening diseases. p-Nitrophenol (PNP) is one of them which are widely used in various industries like pharmaceutical, wood, petroleum, paper-making, textile, dyes, pesticides and many more. The nitro group attached to the p-position of the phenol ring is highly toxic and has harmful effects on the health of various living beings^10,11^. Therefore the development of novel and innovative materials with extremely good sensing property of PNP becomes crucial for their domestic as well as industrial applications. In the past decades, different types of sensors have been assembled for the sensing of PNP. Rahman *et al*., studied the sensing of PNP using Ag_2_O–CNT NCs electrode having the linear concentration range (LDR) of 1.0 nM–0.01 mM^12^. Liu *et al*., fabricated an electrochemical sensor for PNP detection using nanoporous gold, which was able to produce a pair of redox peak for the reversible redox reaction of PNP^13^. Madhu *et al*., utilized biomass-derived activated carbon for the sensing of PNP, with good selectivity and sensitivity^14^. Among the various sensing materials, silver decorated graphene is highly claimed because of its large surface area, high functionality, high sensitivity, small size, easy synthesis procedure, low cost, and low power consumption.

Similarly, energy-storage devices are also in great demand due to the rising energy crisis. To fulfill the increasing energy demand the development of cost-effective storage devices with high energy backup is required^15,16^. AgGr composites are one of them which can be used in supercapacitors with high power densities and fast charge-discharge capabilities. Bello *et al*. fabricated AgNPs decorated 3-D graphene *via* CVD technique for supercapacitor applications. The composite was able to obtain the capacitance of 110 Fg^−1^ ^17^. Dhibar *et al*., fabricated (Ag-PANI)/MWCNTs nanocomposite for high-performance supercapacitor electrodes with the specific capacitance of 528 Fg^−1^ ^18^. Tang *et al*., prepared AgNPs decorated polyaniline/multiwalled super-short carbon nanotubes for supercapacitor application. The composite also showed an excellent electrical conductivity of 18.5 Scm^−1^ ^19^. Sawangphruk *et al*. obtained an extremely high specific capacitance of 828 Fg^−1^ with the AgNP-PANI-graphene/CFP electrode. The as-prepared fully charged supercapacitor was able to power a 3-V motor for 7.30 min^20^. The above-mentioned works of literature show various AgNPs/graphene composites with superior sensing as well as super capacitive properties. The exclusive properties of AgNP/graphene composites have recently increased the huge interest of the researchers to develop easy and facile synthesis techniques and to use these composites in the field of energy storage and sensor technology. In the above-mentioned research, different composites have been used for the sensing of PNP but the sensing of PNP using biomass-derived AgNPs decorated graphene is not shown yet. So, it would be a great achievement to study and understand the sensing of PNP using the AgNPs decorated graphene electrodes. Therefore, the current research work mainly focuses on i) the biomass synthesis of AgGr sample using onion as raw material, ii) investigation of the different properties of the as-synthesized AgGr samples, iii) investigation of the voltammetric sensing of PNP using the AgGr samples synthesized at different process temperature and iv) investigation of the supercapacitive behavior of AgGr electrodes in 1 M H_2_SO_4_.

## Results and Discussion

### Morphological analysis of AgGr samples by AFM and SEM

**Figure 1:**
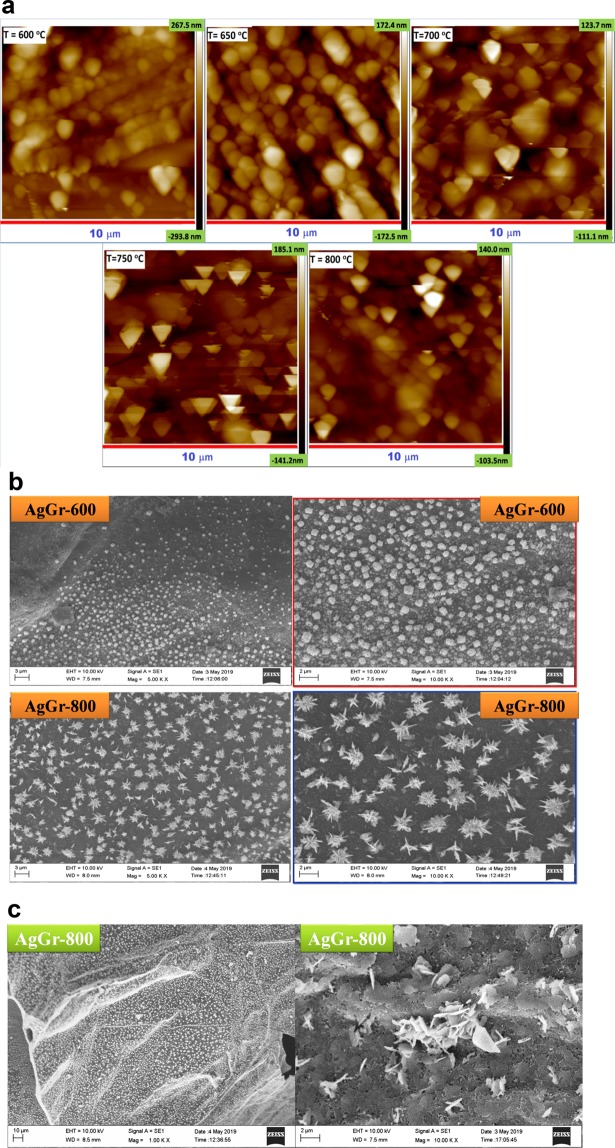
(**a**) AFM images of AgGr samples with the process temperature varying from 600 to 800 °C. (**b**) SEM images of AgGr-600 and AgGr-800 samples showing the structures of AgNPs. (**c**) SEM images of AgGr-800 showing the porous nature of the graphene sheet containing silver nanosheet.

Fig. 1(a) shows the AFM images of AgGr samples with the process temperature varying from 600 to 800 °C. The AFM image shows that Ag nanoparticles are well distributed over the graphene sheet. At lower temperatures of 600 and 650 °C, Ag nanoparticles are highly dense and are distributed on the top layer of the graphene sheet, but at higher temperatures i.e., 800 °C, it is apparent that the Ag particles are incorporated inside the sheet of graphene. The incorporation of AgNPs takes place during the exfoliation process of onion peels. At higher temperatures, the functional groups present in onion peels are removed. Hence, the remaining sheet becomes porous and contains a number of defect sites which are then filled by the AgNPs. The average AgNPs size is in the range of 300 to 600 nm for AgGr-600 while that of AgGr-800 is in the range of 650 to 900 nm as obtained by the AFM images shown in Fig. 1(a). The increase in particle size with the rise in process temperature from 600 to 800 °C is due to the agglomeration of AgNPs to form bigger sized clusters at high temperature due to the formation of more number of Ag-Ag bonds between the Ag atoms. Fig. 1(b) shows SEM images of AgGr-600 and AgGr-800 samples. For AgGr-600 sample no flower-like structure is obtained but a cluster of spherically shaped AgNPs are well spread on the graphene sheet surface with an average particle size of ~100 nm and cluster size of ~500 nm. Interestingly, for AgGr-800 the AgNPs resulted in the formation of the well-dispersed and homogeneous flower-like structure of properly arranged and loosely packed silver nanosheets. Also, it is understandable from the SEM images that at the lower process temperature of 600 °C, the number of AgNPs is dispersed on the surface of the graphene sheet, whereas for AgGr-800 the silver nanoflakes are embedded inside the graphene sheet making it more porous in nature. It is obvious from the SEM images that temperature induces the formation of silver nanosheets, some of which get incorporated inside the graphene sheet as shown in Fig. 1(c) while some reconstruct itself into a flower-like pattern.

### Structural characterization of AgGr samples by XRD and Raman

**Figure 2:**
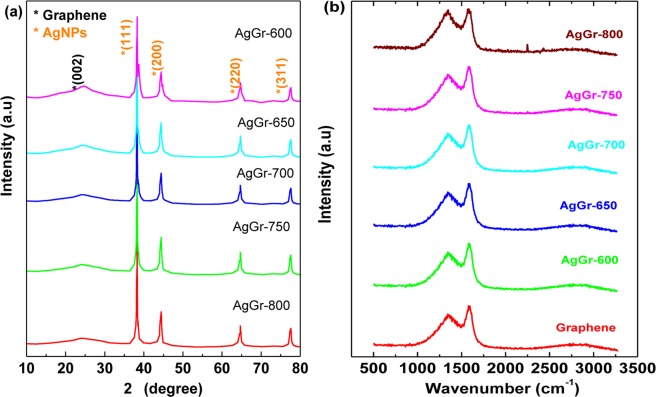
(**a**) XRD patterns and (**b**) Raman spectrum of AgGr samples with varying the process temperature from 600 to 800 °C.

Fig. 2(a) shows the XRD spectra of AgGr samples with the process temperature varying from 600 to 800 °C. The 2θ diffraction pattern observed at 24.47° with the corresponding plane of (002) is assigned for the presence of the graphene sheet. Bhujel *et al*., obtained a similar XRD pattern for graphene at ~24.3° and for Ni decorated graphene samples^21^. Moreover, 2θ diffraction patterns for AgNPs were observed at 38.29, 44.43, 64.67 and 77.59° with the corresponding planes of (111), (200), (220) and (311), respectively. This confirmed the formation of a face-centered cubic structure of AgNPs (JCPDS no 04-0783)^22^. The appearance of XRD peaks for graphene and AgNPs also confirms the successful synthesis of silver decorated graphene. The presence of a sharp peak at 38.29°confirms the good crystallinity of AgNPS. The value of d increased from 3.6 to 3.7 Å as the process temperature was raised from 600 to 800 °C. The opening of interlayer spacing is caused due to the incorporation of more number of AgNPs between the graphene sheets which further leads to the rupture of various sp^2^ hybridized ring structure of C bonds due to the bonding of ring C atom with the AgNPs. The crystallite size of AgNPs is ~35.2 nm for all AgGr samples processed at different temperatures. From the XRD analysis, it is confirmed that variation in process temperature from 600 to 800 °C has no such influence on the crystallite size of AgNPs. The study of Raman scattering is important because the Raman scattering affects the Raman peaks of graphene when AgNPs are incorporated inside graphene layers. Fig. 2(b) displays the Raman spectra of as-deposited graphene and AgGr samples synthesized at a varying process temperature of 600 to 800 °C. The prominent Raman signature peaks observed at 1342.43, 1593.27 and 2767.43 cm^−1^; 1342.17, 1592.99 and 2767.01 cm^−1^; 1341.92, 1592.71 and 2766.59 cm^−1^; 1341.66, 1592.42, 2766.17 cm^−1^; 1341.41, 1592.14 and 2765.75 cm^−1^; and 1348.33, 1583.28 and 2760.75 cm^−1^ correspond to the presence of D, G and 2D bands for graphene, AgGr-600, AgGr-650, AgGr-700, AgGr-750 and AgGr-800, respectively. D band denotes the breathing mode of K-point phonons with A1g symmetry when defects are present either at the basal plane or the edges^23^. G band arises as a result of the doubly degenerate in-plane transverse (iTO) and longitudinal (iLO) optical phonon modes which signify the vibrations of carbon atoms with sp^2^ hybridization. The overtone of D band is recognized by the 2D band which arises when iTO having opposite vectors lead to inelastic scattering near the K-point (zone boundary)^24^. There is an increase in I_D_/I_G_ value when Ag nanoparticles are incorporated in graphene (0.87) and goes on increasing with the rise in process temperature from 600 to 800 °C. Among the various AgGr samples, the highest value of I_D_/I_G_ was shown by AgGr-800 (1.52). The increase in I_D_/I_G_ value suggests an increase of defect states as the Ag particles are incorporated inside the grapheme layers which may be due to the breaking of sp^2^ carbon structure and its bonding with the AgNPs^25^.

### FTIR analysis of AgGr samples

**Figure 3:**
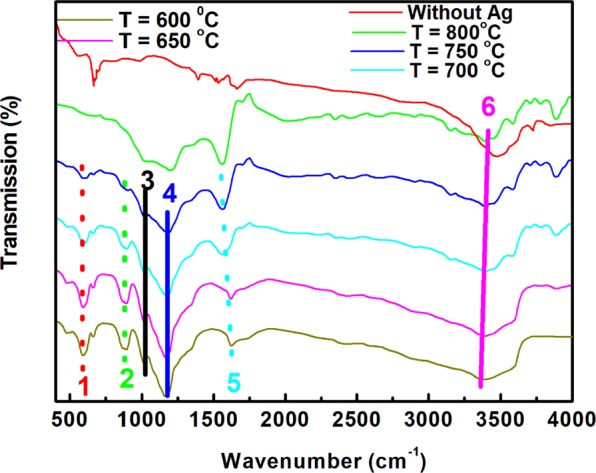
FTIR spectra of AgGr samples with the process temperature varying from 600 to 800 °C.

Fig. 3 shows the FTIR spectra of AgGr sheets with the variation in process temperature from 600 to 800 °C. The FTIR analysis of AgGr samples is required to know the presence of various functional groups present in silver decorated graphene sheets, which is developed during the thermal exfoliation of AgNO_3_ dipped onion peels. The FTIR signature appeared at 3386 and 1399 cm^−1^ are allocated for the –OH stretching vibrations. The presence of a strong IR band at 1598 cm^−1 ^contributes for the stretching vibration of the C=C bond. The signature for C=C becomes more intense when the process temperature is increased from 600 to 800 °C, confirming the presence of more number of C=C bonds for the removal of oxygen and sulfur-containing functional groups from the onion peel during the exfoliation process. The presence of an FTIR signature at ~1224 cm^−1^ is assigned to the presence of an O-O-H bond. Moreover, a small peak obtained at 1010 cm^−1^ for only AgGr samples confirm the appearance of the C-O-Ag bond, which confirms the successful formation of AgGr samples. A similar FTIR band was also obtained by Jiang *et al*. for Ag/graphene composite synthesized for the antibacterial properties^26^.

### BET surface area analysis of AgGr samples

**Figure 4:**
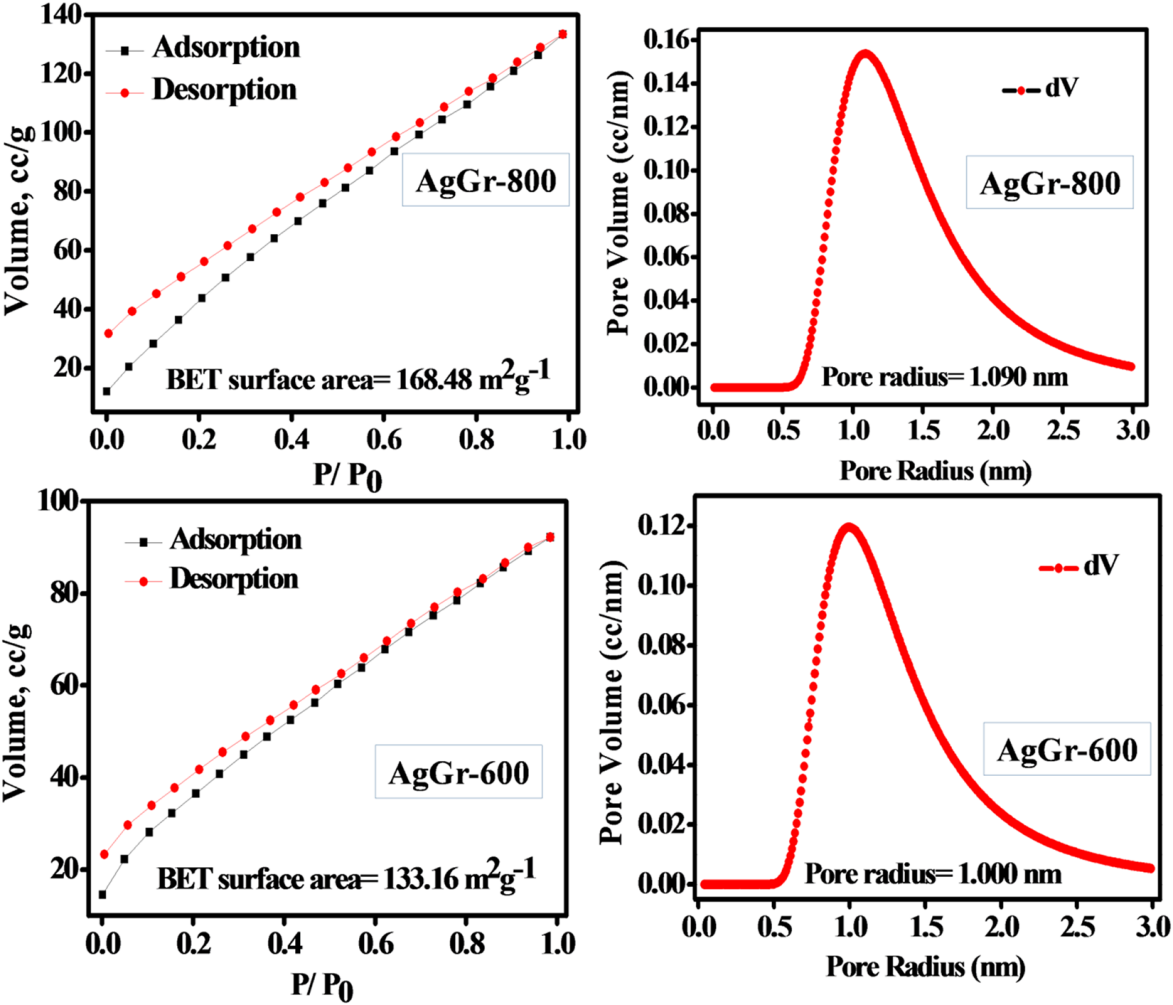
BET N_2_ adsorption-desorption isotherm and pore size distribution curve for AgGr-800 and AgGr-600.

Fig. 4 shows the N_2_ adsorption-desorption isotherm and the pore size distribution curves for AgGr-600 and AgGr-800 at a temperature of 77 K. The BET isotherms obtained for both the samples contain type IV isotherms^27^ with a hysteresis loop over the whole range of relative pressures $(\frac{P}{{P}_{o}})$. The low adsorbed volume at a very low partial pressure for both the AgGr sample confirms the mesoporous structures of the material, as observed by the hysteresis loop. The maximum volume was absorbed by the AgGr-800 sample as compared to AgGr-600, as obtained from the BET isotherm. Moreover, it was observed that the BET surface area for AgGr-800 is 168.48 m^2^g^−1^ with a pore radius of 1.09 nm, while that of AgGr-600 is 133.16 m^2^g^−1^ having the pore radius of 1 nm. The high surface area of AgGr-800 and presence of higher porosity is due to the thermal reduction of maximum numbers of the functional groups attached to the AgGr sheets. Also, the pore size of AgGr-600 is slightly smaller due to the presence of a maximum number of AgNPs on the surface of the graphene sheet. Therefore, the decoration of maximum numbers of AgNPs on the graphene sheet reduces the BET surface area as well as the porosity of the material.

### Electrochemical sensing of PNP using AgGr samples modified glassy carbon electrode

**Figure 5:**
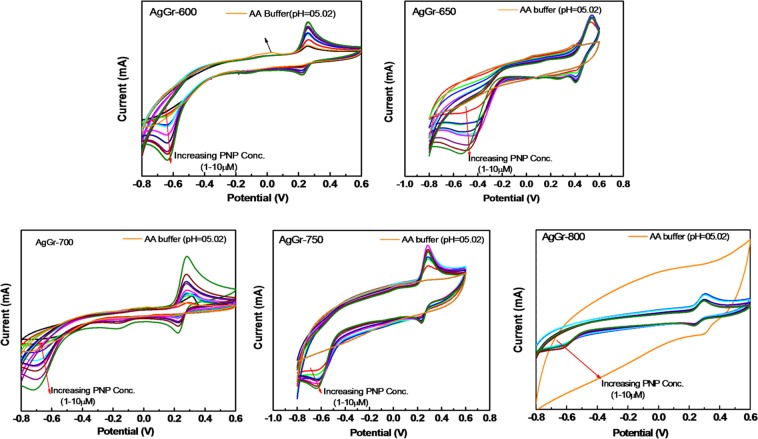
CV curves of AgGr/GCE electrodes at different concentrations of PNP, varying from 1–10 μM.

**Scheme 1:**
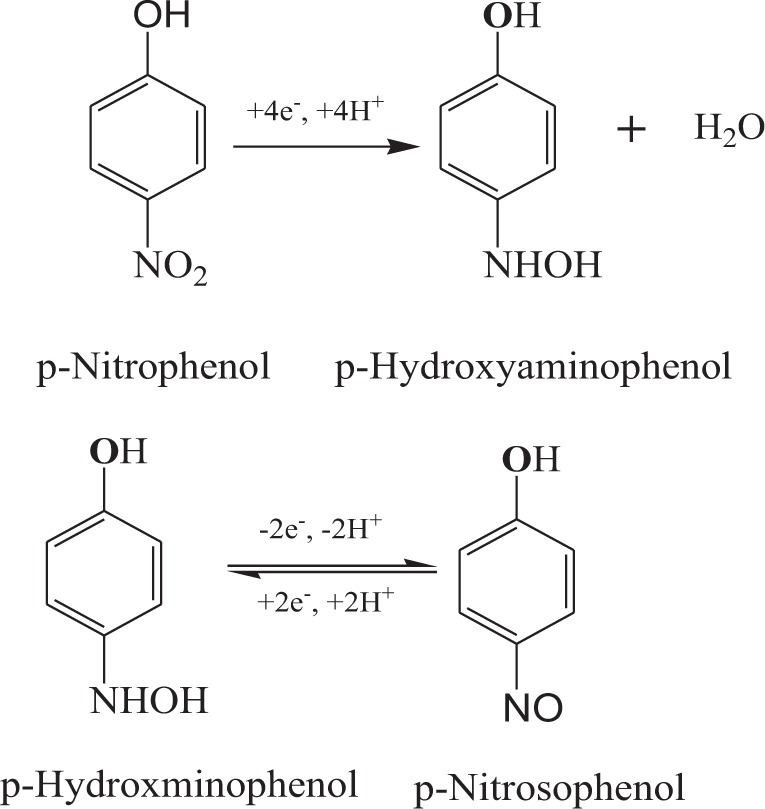
Mechanism for the redox peaks appearing during PNP sensing by AgGr/GCE.

The electrochemical response of PNP by AgGr sample modified glassy carbon electrode (GCE) was studied with the help of cyclic voltammetric (CV) curves as shown in Fig. 5. The electrochemical behavior of AgGr/GCE samples was monitored by the presence of oxidation-reduction peaks in the CV curves, induced by the electroactive surface of AgGr samples. The CV analysis of acetic acid buffer (0.2 M, pH = 5.02) showed no peaks for AgGr/GCE electrodes, indicating the absence of oxidation-reduction phenomenon in buffer solution. However, the addition of 1 μM PNP induced the electrochemical response of AgGr samples. The appearance of a pair of redox peaks at 0.22 V(R1) and 0.26 V(O1) and an irreversible oxidation peak at −0.6 V(R2) for PNP indicates, a three-step electrochemical process undergoing on the surface of AgGr-600/GCE. Kumar *et al*., obtained a reversible PNP oxidation (O1), reduction (R1) peaks at 0.12 and 0.04 V also an irreversible reduction peak (R2) at −0.69 V with MnO NPs/BCA/gold electrode^28^. The redox mechanism of PNP on AgGr/GCE can be explained in Scheme 1 as provided in the earlier literature by Liu *et al*.^29^. The appearance of a large redox peak at ~−0.6 V is due to the reduction of PNP to p-hydroxyaminophenol, whereas the presence of redox peaks at the high potential region is a result of the oxidation of p-hydroxyaminophenol to p-nitrosophenol and the successive reverse reaction to p-hydroxyaminophenol. As shown by Fig. 5 the reduction peak current of PNP obtained at ~−0.6 V decreases with the rise in process temperature. The peak current of PNP at AgGr-800/GCE reduced many times as compared to the AgGr-600/GCE sample.

### Effect of concentration on sensing of PNP

**Scheme 2:**
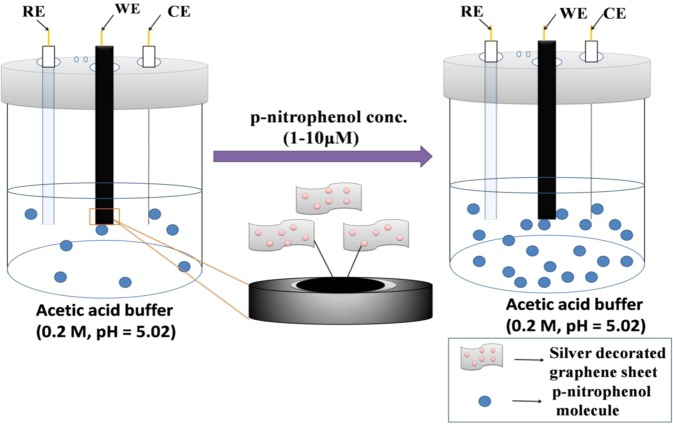
Schematic showing the effect of concentration on the sensing of PNP by AgGr/GCE.

Fig. 5 clearly shows that with an increase in PNP concentration from 1 to 10 μM/L, the area as well as the peak intensity of the CV curve increases. The current response of PNP rises up with the increase in solution concentration as the total volume of the solution containing 1 mole of p-nitrophenol molecule increases, which are capable to reach out to the electrodes required for the sensing purpose. Moreover, it is a well-known fact that the more the number of active particles present in electrolyte, the more is the conductivity and hence more is the current response. This phenomenon is well explained in Scheme 2.

### Effect of process temperature of AgGr samples

It is observed from Fig. 5 that process temperature for the synthesis of the AgGr sample has also a great impact on the sensing of p-nitrophenol. With the rise in process temperature from 600 to 800 °C, the area under the CV curve increases drastically with the reduction of PNP redox peaks signifying an increase in the number of defects states. The electrochemical properties of PNP are highly affected by the surface morphology and the functionalization of AgGr samples. For higher temperature processed samples (*i.e.*, AgGr-750, AgGr-800), the PNP sensing redox peaks are depressed due to the excessive opening of the CV curve due to the increase in double-layer capacitance of graphene sheets with the electrolyte. AgGr-600/GCE sample obtained the most prominent as well as more defined redox peaks for PNP. This is maybe due to the presence of a maximum number of active sites containing AgNPs functional groups available on the surface of AgGr-600/GCE samples. The increment in the number of active sites allows a number of PNP molecules to contact with the surface of AgGr electrodes. Moreover, it was observed from the AFM images that at a lower temperature the AgNPs are well distributed on the surface of the graphene layer, with the smaller sized particle size. This allows maximum active area on the surface of the graphene sheet to make proper contact with the PNP molecules. This could be one of the reasons for the improved PNP sensing by the lower temperature synthesized AgGr samples.

### Effect of scan rate on sensing of PNP

**Figure 6:**
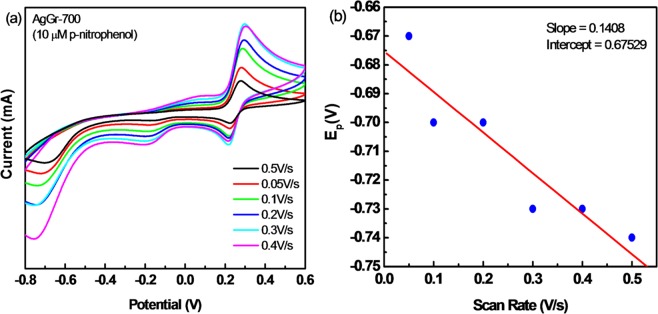
(**a**) CV curve for AgGr-700/GCE plotted at different scan rates. (**b**) Peak potential *vs.* scan rate plot for AgGr-700.

To understand the proper mechanism of PNP sensing with AgGr/GCE electrodes, a detailed study was done by varying the scan rate from 0.05 V/s to 0.5 V/s. It is observed from Fig. 6(a) that the current response increases for all the redox peaks with a rise in the scan rate. To analyze whether the R2 redox reaction is reversible or not, a linear relationship was obtained for the E_p_ versus log $\nu \,$plot, indicating the R2 process to be an irreversible reduction process. To analyze the number of electrons participating in the irreversible redox reaction, a Tafel analysis was done as shown in Fig. 6(b). The calculated Tafel slope (b) comes out to be 0.1408 V confirming the involvement of a single electron in the rate-determining step of R2. The value of α was found out to be 0.422 and the value for αnα was 1.45. The analysis of Eq. (6) showed that a total of four electrons participated in the redox reaction. By solving the Randles–Sevcik Eq. (7) for the reversible redox reaction, it was found that the pair of electrons participated during the oxidation and reduction process of p-nitrophenol. Hence, it was clear from the above analysis that at first 4 e- are involved in the reduction of p-nitrophenol to p-hydroxyaminophenol, followed by a coupled redox reaction with the participation of a pair of electrons, indicating the reversible oxidation and reduction of p-hydroxyaminophenol to p-nitrosophenol, and vice-versa.

### Linear sweep voltammetry of AgGr samples for PNP sensing

**Figure 7:**
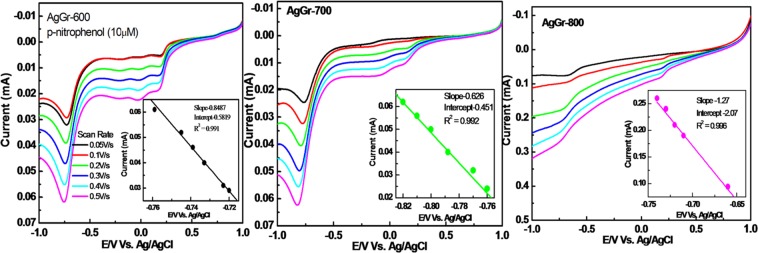
LSV curve for (**a**) AgGr-600/GCE, (**b**) AgGr-700/GCE and (**c**) AgGr-800/GCE.

Linear sweep voltammetry (LSV) is highly sensitive towards the concentration of electrolyte and scan rate. In this report, LSV is performed to study the effect of PNP concentration and scan rate using different AgGr modified GCE. Fig. 7 shows the LSV response curves of AgGr-600/GCE, AgGr-700/GCE and AgGr-800/GCE electrodes for PNP reduction in the presence of acetic acid buffer (0.2 M, pH = 5.02) with varying the scan rate from 0.05 to 0.5 V/s. A sharp reduction peak is observed at ~0.6 V for every AgGr/GCE sample at a scan rate of 0.05 V/s. A consecutive increase in the reduction peak was observed as the scan rate increases from 0.05 to 0.5 V/s. The results are in accordance with the CV curves as shown earlier in Fig. 5. The LSV curve equation for AgGr-600/GCE, AgGr-700/GCE and AgGr-800/GCE are y = 0.848x−0.581, R^2^ = 0.991, y = 0.8626x − 0.451, R^2^ = 0.992 and y = 1.27x − 2.07, R^2^ = 0.996. A significant and well defined linear relationship between peak potential and the peak current is obtained between the scan rates of 0.05 to 0.5 V/s. At first, the PNP molecules are adsorbed on the surface of AgGr/GCE electrodes and then diffuse through the electrodes due to the porous structure of the AgGr samples. The value of R^2^ is 0.996 for AgGr-800/GCE, which also confirms the maximum numbers of pores are available on the surface of the AgGr-800 sample. Hence, it can be concluded that at a higher temperature number of defect sites are generated on the surface of AgGr samples, due to which the PNP sensing becomes weaker due to the excessive opening of the CV curve^30^.

### Electrochemical impedance spectroscopy (EIS) of AgGr samples

**Figure 8:**
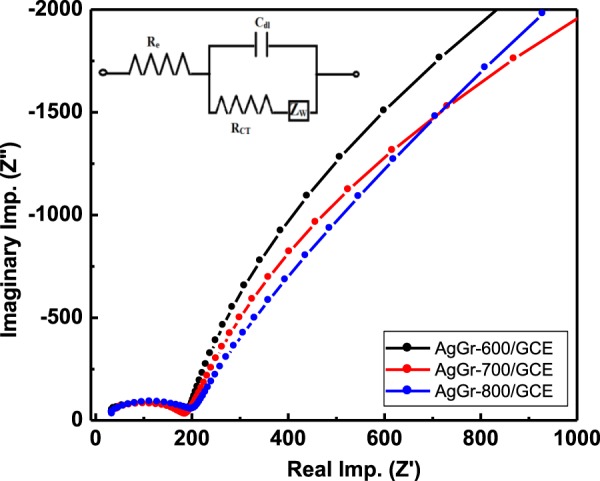
Nyquist plot of AgGr-600/GCE, AgGr-700/GCE, and AgGr-800/GCE.

EIS study was performed in order to understand the interfacial properties of AgGr modified GCE. Fig. 8 shows the Nyquist plots of AgGr-600, AgGr-700 and AgGr-800 modified GCE (where X and Y-axis denote real impedance (Z’) and imaginary impedance (Z”), respectively). The experiment was performed at a frequency in the range of 1–10^6^ Hz and potential amplitude of -1 V. The Nyquist plots of AgGr samples show a semicircular curve ended with a straight line. The semicircle denotes charge transfer resistance (R_CT_) of AgGr modified GCE while the linear part of the Nyquist plot corresponds (Z_w_) which is the Warburg impedance for the finite diffusion of reactants. The Nyquist plot explains a relationship between the charge transfer resistance (R_CT_) and its charge transfer across the AgGr/GCE electrode surface. It is observed from Fig. 8 that the value of R_CT_ is 151.01, 149.56 and 128.5 Ω for AgGr-600, AgGr-700, and AgGr-800, respectively. The minimum value of R_CT_ is obtained AgGr-800/GCE, indicating low charge transfer resistance of AgGr-800/GCE. Moreover, the sharp linear diffusion curve indicates a fast diffusion of reactants through the electrodes. The good conductivity and rapid diffusion of reactants are one of the most important factors that AgGr-800/GCE has greater CV area which depresses the sensing capability of PNP as compare to other electrodes. Govindasamy *et al*. obtained almost similar values of R_CT_ for silver/graphene nano-ribbons nano-composite electrodes^31^.

**Figure 9:**
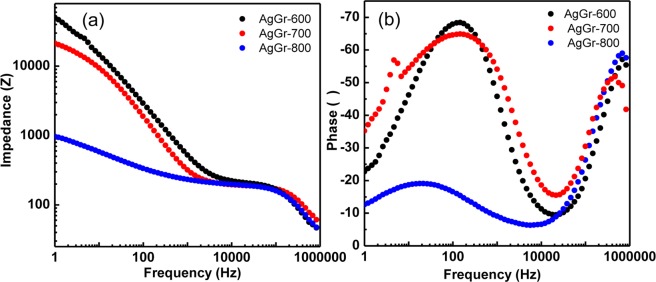
(**a**,**b**) Bode plot of AgGr-600/GCE, AgGr-700/GCE and AgGr-800/GCE.

Fig. 9(a,b) show the impedance *vs.* frequency and phase *vs.* frequency of the Bode plot for AgGr-600, AgGr-700, and AgGr-800 nanocomposites. The value of impedance is 50742.08, 20854.88 and 944.2 Ω with the corresponding phase angle of 34.58, 22.39 and 12.69 °, for AgGr-600, AgGr-700 and AgGr-800, respectively obtained at lower frequency region (10^1^–10^2^ Hz) corresponds to the Warburg impedance. The maximum impedance was observed for the AgGr-600 sample which confirmed the higher diffusion resistance offered by the AgGr-600/GCE electrode which inhibits the ease of flow of PNP molecules through the pores of the electrode. The value of impedance at the higher frequency region of ~10^6^ Hz and φ = φ_max_(−56.86, −51.7 and −58.57) for AgGr-600, AgGr-700 and AgGr-800, respectively give the values of electrolytic resistance (R_e_). Moreover, the value of impedance at the frequency range of (10^3^ to 10^4^ Hz) with the corresponding phase angle of (φ = 0°) is assigned for the total resistance offered by the electrode and the electrolyte i.e. R_e_ + R_CT_. The estimated values of R_CT_ are 140.6, 150.3 and 154.8 Ω for AgGr-800, AgGr-700, and AgGr-600, respectively. From the Bode plot as well, it was confirmed that minimum charge transfer resistance was observed for AgGr-800/GCE electrode which is capable of giving the higher value of capacitance but is least important for the sensing purpose due to the excessive opening of CV curve and the suppression of PNP redox peak at −0.6 V.

### Evaluation of supercapacitive performance of AgGr samples in 1M H_2_SO_4_

**Figure 10:**
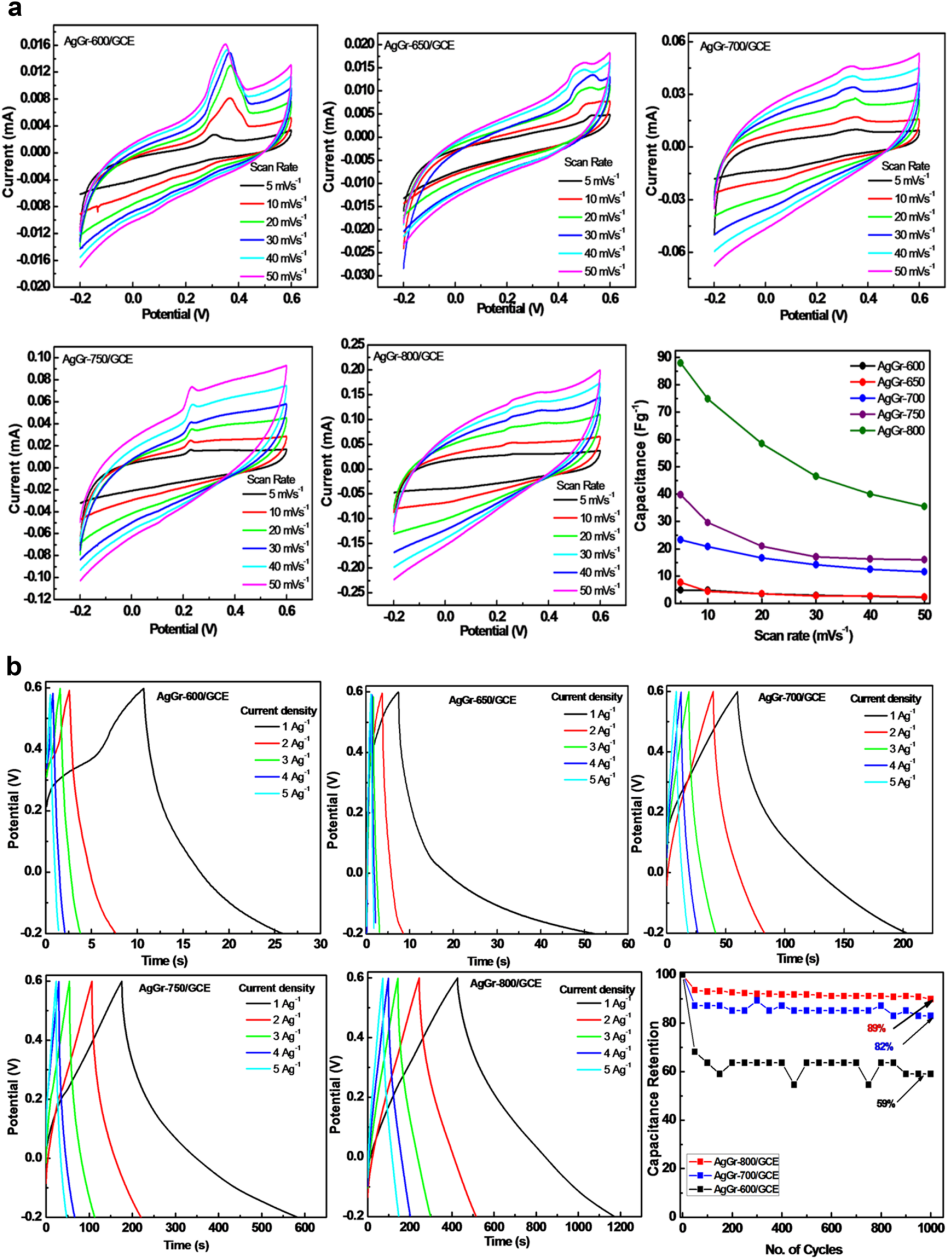
(**a**) CV curves and capacitance versus scan rate plot of AgGr/GCE in 1 M H_2_SO_4_. (**b**) GCD curves of AgGr/GCE at different current densities and capacitance retention of AgGr/GCE samples.

Fig. 10(a) shows the cyclic voltammetric curves of AgGr/GCE by taking 1 M H_2_SO_4_ aqueous solution as an electrolyte. A distorted CV curve was obtained for all the AgGr/GCE with the appearance of a redox peak at around ~0.35 V for AgGr-600 and ~0.25 V for AgGr-800, which attributes the pseudocapacitive behavior of AgGr samples due to the redox activity of Ag to Ag^+^. The area of CV curve was increased from AgGr-600 to AgGr800 which may be caused as a result of the quality improvement of the graphene sheet and also due to the increasing porosity of the sheet. Moreover, it is clear from the SEM images that at higher process temperatures the AgNP flakes are well distributed over the graphene sheet also some of them are incorporated inside. The CV analysis was done by varying the scan rates from 5 to 50 mVs^−1^, and within the potential range of −0.2 to 0.6 V. From the CV curves of AgGr/GCE, it was observed that maximum capacitance was obtained at the scan rate of 5 mVs^−1^. Therefore, by considering 5 mVs^−1^ as the scan rate, the CV analysis was done and was observed that the value of specific capacitance increased from 1.3 to 88 Fg^−1^ as the process temperature was increased from 600 to 800 °C. Maximum capacitance was observed for AgGr-800/GCE due to the reason as explained earlier. To study the supercapacitive behavior of AgGr/GCE, CV analysis was further carried out at different scan rates varying from 5 to 50 mVs^−1^. A noticeable increase in the area of CV curve was observed at higher scan rates but at the same time, the specific capacitance decreased. This can be explained as the poor electronic interaction between the electrode and the electrolyte material at higher scan rates. The value of specific capacitance obtained for different AgGr/GCE with the varying scan rates is also shown in Fig. 10(a).

To confirm the supercapacitive properties of AgGr/GCE, galvanostatic charge-discharge (GCD) measurements were done at different current densities as shown in Fig. 10(b). The appearance of a triangular-shaped GCD curve illustrates a good capacitive property of AgGr/GCE. The calculated values of specific capacitance were 1.9, 5.6, 17.8, 51.5 and 93.5 Fg^−1^ for AgGr-600/GCE, AgGr-650/GCE, AgGr-700/GCE, AgGr-750/GCE, and AgGr-800/GCE, respectively, at the current density of 1 Ag^−1^. From both of the CV and GCD analysis, the maximum capacitance was obtained by AgGr-800/GCE. The improvement of graphene sheets as well as excellent bonding between the AgNPs and graphene sheet, facilitates a rapid electron transfer between the electrolyte and the electrode materials resulting in an increase in the specific capacitance value. The calculated values of specific capacitance at different current densities are inserted in Table 1. Moreover, from the capacitance retention curve as shown in Fig. 10(b) it is clear that maximum capacitance retention was obtained for AgGr-800/GCE at a current density of 1 Ag^−1^. The AgGr-800/GCE sample was stable even after the 1000^th^ cycle with the retention of 89% of the initial capacitance. Minimum retention of just 59% was obtained by the AgGr-600/GCE sample due to its poor capacitive property. Hence, the retention analysis of AgGr/GCE samples displayed that better cycling stability was obtained by AgGr-800/GCE, than AgGr-700/GCE followed by AgGr-600/GCE in 1 M H_2_SO_4_.

**Table 1:** The calculated values of specific capacitance at different current densities for all AgGr/GCE electrodes processed at different temperatures.

Sample	Specific capacitance in (Fg^−1^) at the current densities of				
	1 Ag^−1^	2 Ag^−1^	3 Ag^−1^	4 Ag^−1^	5 Ag^−1^
AgGr-600/GCE	1.9	1.23	0.78	0.65	0.46
AgGr-650/GCE	5.56	1.2	0.56	0.45	0.5
AgGr-700/GCE	17.8	10.8	8.45	7.0	6.1
AgGr-750/GCE	51.5	28.5	21.8	18.1	15.3
AgGr-800/GCE	93.5	67.0	58.2	52.3	47.2

### Corrosion analysis of AgGr samples in H_2_SO_4_

**Figure 11:**
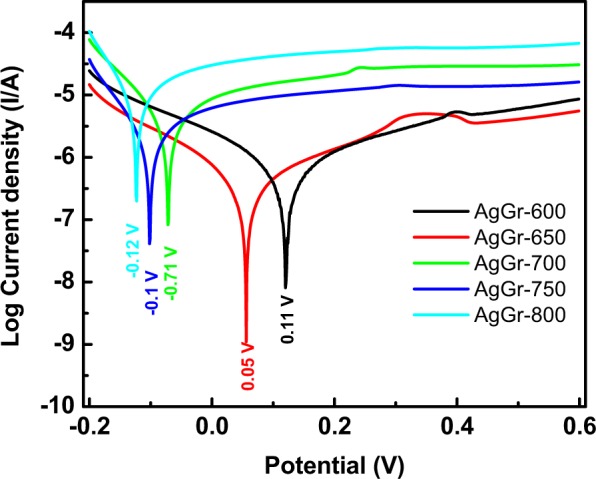
Tafel plot of AgGr/GCE in 1 M H_2_SO_4_.

Fig. 11 shows the Tafel plots of AgGr/GCE samples in the 1 M H_2_SO_4_ solution. It is very interesting to monitor that AgGr-600/GCE has the most positive value of corrosion potential (0.11 V), while that of AgGr-800/GCE has the most negative potential value (−0.12 V). The shifting of the corrosion potential towards the negative side is due to the active corrosion reaction taking place at the coating/substrate surface. AgGr-800/GCE is likely to degrade easily as compare to lower temperature processed AgGr samples. The formation of silver flakes and increasing porosity of the graphene sheet allows the formation of more active sites in AgGr-800/GCE, which facilitates easy interaction between the coating and the corrosive solution. While in the case of AgGr-600/GCE, the presence of less cavity and holes prevents the adsorption of ions that are responsible for corrosion.

## Conclusion

A new, easy and green method is utilized for producing silver decorated graphene for the voltammetric detection of p-nitrophenol (PNP). The biomass-derived silver decorated graphene (AgGr) samples are prepared using an APCVD reactor with varying the process temperature from 600 to 800 °C. The SEM image shows the distribution of the flower-like structure of Ag flakes in the graphene sheet for AgGr-800 sample while for AgGr-600 sample, the nanoparticles agglomerates to form bigger sized clusters of AgNPs. The interlayer spacing and I_D_/I_G_ ratio of the AgGr samples varied from 3.6 to 3.7 Å and 0.87 to 1.52 as the process temperature was raised from 600 to 800 °C. Also, the increased number of active sites on the surface of AgGr-800, the presence of a higher number of defects makes it least useful for the sensing of PNP due to the excessive opening of the CV curve. Although all AgGr samples are capable of PNP sensing, AgGr-600 are extremely suitable for the sensing of PNP, owing to its high surface to volume ratio due to smaller sized silver nanoparticles and synergistic effect between the AgNPs and graphene sheet, present on the surface of the graphene sheet. The specific capacitance of AgGr/GCE varied from 1.9 to 93.5 Fg^−1^ as the process temperature was increased from 600 to 800 °C. Hence, this novel method can be used for the large scale production of various metal decorated graphene samples for their application in different fields.

## Methods

### Synthesis of AgGr samples

**Scheme 3:**
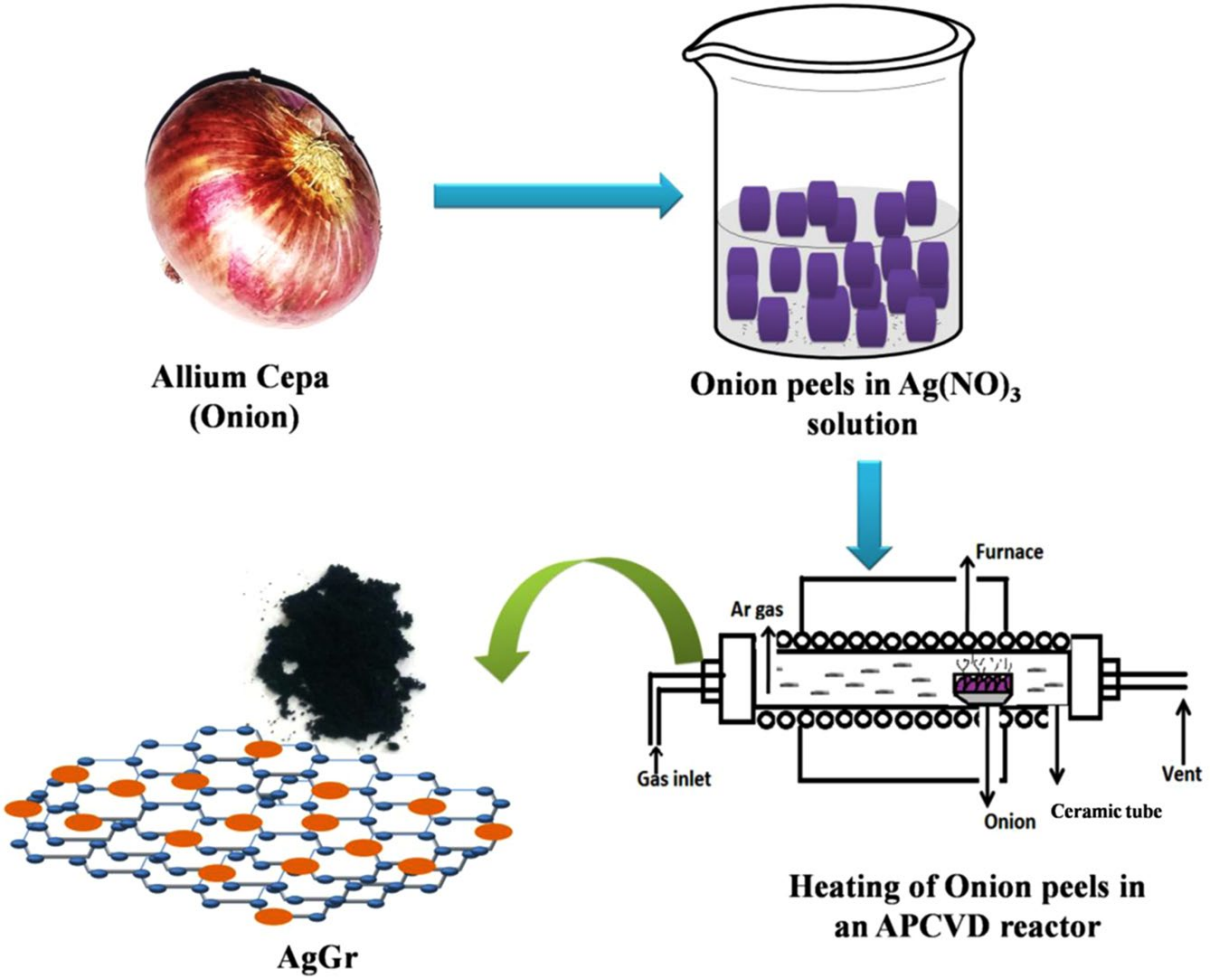
The whole schematic for the synthesis of silver decorated graphene samples.

Onion peels were extracted from fresh onion (Allium cepa) which is utilized as a raw material for the synthesis of Ag decorated graphene. The extracted onion peels were at first dipped in an aqueous solution of silver nitrate for 12 h. After that a temperature of 110° was employed for around 3 h to remove the moisture from the AgNO_3_ dipped onion peels. The AgNO_3_ dipped onion peels were then subjected to a silicon carbide resistance vacuum furnace (made: V.B. Ceramics) for the exfoliation of onion peels under anaerobic condition. To maintain an inert atmosphere inside the furnace, Ar gas was purged during the heating process. The successive quality production of AgGr samples were achieved at the temperature of 600 to 800 °C and 5 °C per minute ramp rate. The same temperature was fixed and maintained the same for 2 hrs. After that, the samples are taken out dried in a desiccator and sent for the characterization. The like approach was applied by Bhujel *et al*. for the synthesis of Ni decorated graphene and also for graphene^21,32^. The whole synthesis procedure of AgGr samples is shown in Scheme 3.

### Characterization of AgGr samples

The AFM characterization of AgGr samples was done by (Nanoscope III scanning probe microscope) and SEM characterization was done by (EVO MA18 with Oxford EDS(X-act)). The XRD characterization was done with the help of (PAN Analytical Spectris Technologies (PW 3040/60)) and Raman characterization was done by (WITEC ALPHA 300 RS by 532 nm laser at 1.5 mW power). The FTIR characterization was done by (SCHMIDZU ATR-FTIR spectrophotometer). BET analysis was done using the Quantachrome Nova Touch LX2 surface area and a pore size analyzer. Finally, the electrochemical properties of AgGr samples were analyzed with the help of CH Instruments, Inc. (Electrochemical Analyzer CHI608E) using Ag/AgCl as the reference electrode, the working electrode as AgGr coated glassy carbon electrode and Pt wire for the counter electrode. The electrolytic solution contained 1 M acetic acid buffer (pH = 5.02). Capacitance measurement and corrosion analysis were done in 1 M H_2_SO_4_ solution.

### Structural analysis of AgGr samples

To find out the interlayer spacing (d) between the graphene sheets Bragg’s Eq. (1) was used and crystallite size of AgNPs was calculated using Debye Scherrer’s Eq. (2)

1 $$d=\frac{n\lambda }{2\,\sin \,\theta }$$

2 $${\rm{D}}=0.9\lambda /\beta \,\mathrm{Cos}\,\theta $$

where d refers to the interlayer spacing, θ is to the diffraction angle and **λ** denotes the X-ray wavelength. Bhujel *et al*. used a similar equation for determining the interlayer spacing of iron oxide/RGO composites^33^. D gives the crystallite size of AgNPs and β gives the FWHM of the most intense XRD peak in radians.

### Electrochemical analysis of AgGr samples

Tafel analysis was done using the following equations

3 $${E}_{p=}(\frac{b}{2})log\nu +Constant$$

4 $$b=\frac{2.303RT}{\alpha F}$$

5 $$\alpha n\alpha =\frac{0.048}{{E}_{p}-{E}_{p/2}}$$

6 $${I}_{p}=(2.99\times {10}^{5})n{(\alpha n\alpha )}^{1/2}AC{D}^{1/2}{\nu }^{1/2}$$

7 $${I}_{p}=(2.69\times {10}^{5}){n}^{3/2}AC{D}^{1/2}{\nu }^{1/2}$$

where E_p_ gives peak potential, $\nu$ refers to the scan rate and b gives the Tafel slope. α is the transfer coefficient and T, F, and R represent the temperature in Kelvin, Faraday constant and gas constant, respectively. E_p/2_ is half-height potential. C is the concentration in mole cm^−3^, A is the area in cm^2^ and D is the diffusion coefficient in cm^2^ s^−1^.

The specific capacitance from CV was further calculated by using the equation as shown below

8 $${C}_{m}=\frac{1}{({\rm{m}}{\rm{\nu }}({V}_{b}-{V}_{a})}{\int }_{{V}_{a}}^{{V}_{b}}IdV$$

where ${C}_{m}$ (Fg^−1^) is denoted for specific capacitance, I (A) is current of the CV curve, ${\rm{\nu }}$ (Vs^−1^) refers to the scan rate; m (g) denote the mass of active electrode materials and ${V}_{a}$ and ${V}_{b}$ (V) are a high and low potential range of the CV curve. Using the GCD curves, the specific capacitance was again calculated by applying the equation given below

9 $${C}_{SC}=\frac{I\Delta t}{m\Delta V}$$

where ${C}_{SC}$ refers to the specific capacitance, I denote discharge current, Δ*t* is the discharge time after IR drop, m is the mass of electroactive material and Δ*V* is the potential range.
